# Green extraction of bioactive polysaccharides from *Zanthoxylum bungeanum* using deep eutectic solvents: enhanced yield, antioxidant activity, and functional hydrogel formation

**DOI:** 10.1016/j.fochx.2026.104612

**Published:** 2026-10-13

**Authors:** Mengjia Wu, Guoxing Yu, Wenya Hu, Yulin Liu, Yonghong Liu

**Affiliations:** College of Forestry, Northwest A&F University, Yangling, Shaanxi 712000, China

**Keywords:** *Zanthoxylum bungeanum*, Polysaccharides, Deep eutectic solvent, Antioxidant activity, Hydrogel fabrication

## Abstract

An ultrasound-assisted deep eutectic solvent (DES) method was developed for extracting polysaccharides from *Zanthoxylum bungeanum* pericarps (ZBP) and leaves (ZBLP). A choline chloride–oxalic acid (2:1) DES was selected. Under optimized conditions, yields of ZBP-D and ZBLP-D reached 25.88% and 29.79%, significantly higher than ultrasound-assisted water extraction (6.27% and 12.13%). DES-extracted polysaccharides shared similar structures but showed selectively enhanced antioxidant activity, with significantly lower IC₅₀ values against DPPH and hydroxyl radicals (0.231 mg/mL and 0.513 mg/mL for ZBP-D and ZBLP-D) compared to ultrasound-assisted water-extracted samples, although the latter performed better in the ABTS^+^ assay. The polysaccharides were formulated into hydrogels with dense porous networks, high swelling ratios (∼1200%), high loading ratios (∼69%), antioxidant retention, and hemocompatibility (safe load: 2.0 mg/mL for ZBP-D, 4.0 mg/mL for ZBLP-D). This green DES method enables efficient, bioactive polysaccharide extraction and hydrogel fabrication, providing a sustainable strategy for the value-added utilization of *Z. bungeanum* by-products.

## Highlights

• ChCl-OA DES was optimal among 12 systems for *Z. bungeanum* polysaccharide extraction.
• DES extraction increased yields by 4.13-fold for pericarp and 2.46-fold for leaves.
• DES-extracted polysaccharides showed enhanced DPPH and hydroxyl radical scavenging.
• ZBP-D and ZBLP-D hydrogels exhibited ∼1200% swelling and hemolysis <5%.

## 1 Introduction

*Zanthoxylum bungeanum* (*ZB*) commonly known as Sichuan pepper, is a plant of considerable economic and cultural significance in East Asia. It is esteemed both as a culinary spice and a traditional medicinal herb (Xu et al., 2020). The primary commercial product, the pericarps, is prized for its distinctive numbing sensation and pungent flavor. Notably, the pericarps and leaves, which constitute 20%–30% of the total biomass, are valuable medicinal and edible resources with significant development potential (Meng et al., 2021). Modern pharmacological studies have demonstrated that these components are rich in bioactive compounds, including polysaccharides, volatile oils, and flavonoids, which contribute to their antioxidant, anti-fatigue, and immunomodulatory effects (Gong et al., 2021; Yang, 2008). As materials with both medicinal and edible uses, the comprehensive utilization of *ZB* pericarps and leaves holds considerable promise for value-added applications.

Plant polysaccharides, defined as glycans with a polymerization degree greater than 10, are crucial for normal biological functions and exhibit diverse bioactivities, including antioxidant, anti-infective, and immunomodulatory effects (Yarley et al., 2021; Zou et al., 2020). Studies have shown that polysaccharides from *ZB* leaves (ZBLPs) can alleviate physical fatigue, by regulating glycogen metabolism and enhancing the antioxidant defense system (Zhou & Yang, 2023). Similarly, polysaccharides from *ZB* pericarp (ZBPs) have been shown to possess pharmacological properties such as antitumor, anesthetic, analgesic, antibacterial, insecticidal, and antioxidant effects (Chen et al., 2021; Li et al., 2015). Despite their potential, research on the extraction methods for ZBPs and ZBLPs are limited, especially regarding efficient extraction techniques and detailed structure-activity relationships. Hot water extraction is the most common technique for isolating polysaccharides from plant materials due to its simplicity and low cost, making it ideal for small-scale laboratory or production settings. However, it has drawbacks like long extraction times and high energy consumption (Yim et al., 2021). While enzyme-assisted extraction leverages the advantage of facilitating reactions under mild conditions, its application is often limited by high costs and challenges in scaling up for industrial applications. Furthermore, the introduction of enzymes complicates subsequent purification processes, hindering the preparation of high-purity polysaccharides (Guo et al., 2020).

In recent years, deep eutectic solvents (DESs) have gained attention as a new generation of green and sustainable solvents. Comprising a hydrogen bond acceptor (HBA) and a hydrogen bond donor (HBD), DESs form a homogeneous liquid through an intricate hydrogen-bonding network. Their key advantages include low volatility, biodegradability, low toxicity, and highly tunable physicochemical properties, which depend on the selection of HBA/HBD components and their molar ratios (Farajzadeh et al., 2016; Vian et al., 2017). This tunability enables the targeted extraction of specific bioactive compounds. Due to these characteristics, DESs have been increasingly used to extract various bioactive components—such as polysaccharides (Liang et al., 2019), triterpenic acids (Silva et al., 2020), and polyphenols (Wei et al., 2015)—from diverse plant materials, often achieving higher extraction efficiency and better preservation of bioactivity than traditional methods. However, there are few reports on using DESs to extract polysaccharides from *ZB* pericarps and leaves, and the structure-activity relationships of DES-extracted *ZB* polysaccharides remain largely unexplored.

The primary objective of this work is to develop and optimize a green DES-based extraction method for *ZB* polysaccharides, with a focus on understanding extraction performance, comparing with conventional methods, and evaluating the bioactivity of the recovered polysaccharides. As a secondary and exploratory objective, the extracted polysaccharides were formulated into hydrogels to preliminarily assess their physicochemical properties and hemocompatibility. This hydrogel part serves as a proof-of-concept rather than a comprehensive biomedical validation, and the results are presented to provide fundamental data for potential future development rather than to establish ready-to-use biomedical products.

## 2 Materials and methods

### 2.1 Plant materials and sample preparation

Fresh, mature *ZB* fruits and disease-free leaves were gathered from three healthy ‘Wududahongpao’ plants in Wudu District, Longnan City, Gansu Province, China. The seeds and stems were removed from the pericarps and leaves, which were then washed with running deionized water and shade-dried at ambient temperature (25 ± 2 °C) for 72 h, with the drying duration extended if necessary until no visible surface moisture remained. The shade-drying step was implemented to avoid enzymatic browning and the loss of volatile components that could occur under direct high-temperature drying. These samples were further dried in a blast drying oven at 40 °C until they reached a constant weight, defined as a weight change of less than 0.2 mg between two successive weighings at 2-h intervals. Once dried, the pericarps and leaves were ground into a powder and sifted through a 40-mesh sieve. The resulting powder was stored in vacuum-sealed bags at 4 °C for subsequent use.

### 2.2 Chemical and reagents

Choline chloride (CC, ≥ 98%), oxalic acid (OA, ≥ 98%), DL-malic acid (MA, ≥ 99.5%), and 1,4-butanediol (≥ 98%) were sourced from Shanghai Macklin Biochemical Technology Co., Ltd. (Shanghai, China). Betaine (≥ 99%), D (+)-glucose, D-galacturonic acid monohydrate (GalA, ≥ 98%), D-glucuronic acid (GlcA, ≥ 98%), DL-arabinose (Ara, ≥ 98%), D-galactose (Gal, ≥ 98%), D-mannose (Man, ≥ 98%), D-(+)-xylose (Xyl, ≥ 99%), L-(−)-glucose (Glc, ≥ 98%), and rhamnose (Rha, ≥ 98%) were obtained from Shanghai Yuanye Bio-Technology Co., Ltd. (Shanghai, China). The DPPH, ABTS, and hydroxyl radical scavenging capacity assay kits for water-soluble substances were purchased from Beijing Solarbio Science & Technology Co., Ltd. (Beijing, China). Glycerol (≥ 99%), urea (≥ 99%), anhydrous ethanol (≥ 99.7%), phenol (≥ 99%), concentrated sulfuric acid (H₂SO₄, 95–98%), concentrated hydrochloric acid (HCl, 36–38%), methanol (≥ 99.9%, HPLC grade), acetonitrile (≥ 99.9%, HPLC grade), and glacial acetic acid (≥ 99.5%) were purchased from Guangdong Guanghua Sci-Tech Co., Ltd. (Guangdong, China). Ethylene glycol (EG, ≥ 98%) was supplied by Sichuan Xilong Scientific Co., Ltd. (Sichuan, China). Trifluoroacetic acid (TFA, ≥ 99%) was obtained from Shandong Xiya Chemical Technology Co., Ltd. (Shandong, China). Sodium hydroxide (NaOH, ≥ 96%) was sourced from Sinopharm Chemical Reagent Co., Ltd. (Shanghai, China). 1-Phenyl-3-methyl-5-pyrazolone (PMP, ≥ 99%), pancreatin (porcine pancreas, 4 × USP specifications), chitosan (deacetylation degree ≥95%, viscosity 100–200 mPa·s), and 1× phosphate-buffered saline (PBS, 0.01 mol/L, pH 7.2–7.4) were purchased from Beijing Solarbio Science & Technology Co., Ltd. (Beijing, China). Chloroform (≥ 99%) and potassium bromide (KBr, ≥ 99.5%, IR grade) were sourced from Tianjin Kemiou Chemical Reagent Co., Ltd. (Tianjin, China). Citric acid monohydrate (C₆H₈O₇·H₂O, ≥ 99.5%) was purchased from Shanghai Macklin Biochemical Technology Co., Ltd. (Shanghai, China). Triton X-100 (≥ 98%) was obtained from Beijing Bio-Tech Co., Ltd. (Beijing, China). The reagents used in high-performance liquid chromatography (HPLC) were of chromatographic grade, while all other reagents were of analytical grade. For HPLC analysis, ultrapure water (resistivity ≥18.2 MΩ·cm) was used throughout the experiment; for other general purposes (e.g., sample preparation, DES synthesis, and extraction), deionized water was employed. No other organic solvents were used for extraction or sample preparation beyond those explicitly listed above.

### 2.3 Synthesis and screening of DESs

DESs were synthesized using the heating-stirring method (Fu, Wang, Belwal, Xie, et al., 2021). In this study, choline chloride or betaine served as the HBA. Each HBA was paired with a specific HBD, as outlined in Table 1, and mixed at designated molar ratios. The mixtures were heated and stirred at 90 °C for 3–4 h using a constant-temperature magnetic stirrer until a homogeneous, transparent liquid was achieved, signifying successful DES formation. To address the high viscosity of the synthesized DESs, a specific amount of water was added. The addition of water to hydrophilic DES systems is a well-established strategy to reduce viscosity and improve mass transfer during extraction. Literature reports have demonstrated that the viscosity of DES can be significantly reduced upon water addition, thereby enhancing extraction efficiency (Liu et al., 2019). In the context of DES-based polysaccharide extraction, optimal water contents in the range of 27% to 34% (*w*/w) have been reported in recent optimization studies (Kim et al., 2025; Wen & Chen, 2025; Zhang & Wang, 2017). Given the reported optimal range (27–34%) and the need for a standardized condition for initial screening, 30% (*w*/w) was selected as a reasonable starting point. We acknowledge that a systematic optimization of water content specifically for the *Z. bungeanum* matrix would be beneficial in future studies.

**Table 1:** Chemical composition and molar ratio of HBA and HBD in DES.

DES name	HBA	HBD	Molar ratio
DES1	Choline chloride	Oxalic acid	1:1
DES2	Choline chloride	DL-malic acid	1:1
DES3	Choline chloride	Glycerol	1:2
DES4	Choline chloride	Urea	1:2
DES5	Choline chloride	Ethylene glycol	1:2
DES6	Choline chloride	1,4-butanediol	1:2
DES7	Betaine	Oxalic acid	1:1
DES8	Betaine	DL-malic acid	1:1
DES9	Betaine	Glycerol	1:2
DES10	Betaine	Urea	1:2
DES11	Betaine	Ethylene glycol	1:2
DES12	Betaine	1,4-butanediol	1:2

### 2.4 Extraction and recovery of ZBPs and ZBLPs

Polysaccharides were extracted from the prepared powders using 12 different DES systems with ultrasound assistance. The extractions took place in an ultrasonic instrument (KQ-500E, 40 kHz, 500 W; Kunshan Ultrasonic Instrument Co., Ltd., Kunshan, China). Based on preliminary experiments and previous literature (Nie et al., 2020; Pan et al., 2022), the extraction conditions were set as follows: temperature at 60 °C, duration of 60 min, a solvent-to-solid ratio of 30:1 (mL/g), and ultrasonic power of 500 W. These conditions were used to screen the DESs according to their chemical composition and molar ratio. After extraction, the mixtures were centrifuged at 4000*g* for 20 min to collect the supernatant. The combined supernatant was concentrated using a rotary evaporator to about one-third of its original volume. To remove proteins, pancreatin (porcine pancreas, 4 × USP specifications, Beijing Solarbio Science & Technology Co., Ltd.) was added to the concentrate at a concentration of 5 U/mL, and the solution was incubated at 40 °C for 8 h. Pancreatin was chosen for its mild enzymatic action under physiological conditions, which avoids the use of organic solvents (e.g., chloroform in the Sevag method) or strong acids (trichloroacetic acid) that could cause polysaccharide degradation or introduce toxic residues. Direct radiolabeling evidence demonstrates that plant cell wall polysaccharides are not degraded to low-molecular-weight products, nor solubilized, by pancreatin treatment, whereas contaminating proteins are hydrolyzed to amino acids and oligopeptides (Miller et al., 1995). This selectivity is consistent with recent reviews confirming that enzymatic deproteinization is a viable and broadly applicable strategy for plant polysaccharides (Huang et al., 2021), and that enzymatic methods can achieve high polysaccharide retention rates (>92%) while effectively removing proteins (Li et al., 2019). Although the latter study employed neutral protease rather than pancreatin, together these works support the general principle that protease treatment under mild conditions preserves polysaccharide structural integrity. The enzyme was subsequently inactivated by heating the mixture at 95 °C for 30 min. After cooling to room temperature, the extract was mixed with 4 volumes of anhydrous ethanol and stored at 4 °C overnight to precipitate the polysaccharides. The precipitates were then recovered by centrifugation at 4000*g* for 5 min. The resulting pellets were dissolved in ultrapure water and lyophilized for 48 h to obtain the crude polysaccharides from the pericarp (designated as ZBP-D) and leaves (designated as ZBLP-D), which were stored at 4 °C for further analysis. The total sugar content was determined by the phenol‑sulfuric acid method using glucose as the standard. The standard curve was established as y = 0.0145× + 0.0312 (*R*^2^ = 0.9991). The polysaccharide yield was calculated as the weight percentage of the total extracted polysaccharides relative to the dry weight of the initial raw material (*ZB* pericarp or leaf powder) and is expressed as a percentage (Zhang et al., 2025).

### 2.5 Optimization of the extraction process

The extraction process was optimized using the selected DES. Initially, single-factor experiments assessed the impact of three key variables on polysaccharide yield: extraction temperature (ET: 40, 50, 60, 70, 80, 90 °C), extraction time (ED: 20, 40, 60, 80, 100, 120 min), and solvent-to-solid ratio (SSR: 10, 20, 30, 40, 50, 60, 70, 80 mL/g). The fixed conditions for the single-factor experiments were as follows: ultrasonic power of 500 W, DES1 (choline chloride-oxalic acid, 2:1) with 30% (*w*/w) water content, and a solid-to-liquid ratio of 30:1 (mL/g) when investigating the effects of temperature and time. For the investigation of solid-to-liquid ratio, the temperature and time were fixed at 60 °C and 60 min, respectively. The tested ranges for each variable were: extraction temperature (40–90 °C), extraction time (20–120 min), and solid-to-liquid ratio (10:1–80:1, mL/g). Following this, response surface methodology (RSM) was applied to refine the process further. A three-level, three-factor Box-Behnken design (BBD) was generated using Design-Expert software to assess the interactive effects of the independent variables. Seventeen experimental runs were conducted, as detailed in Supplementary Tables S1 and S2, with polysaccharide yield as the response factor. The goal was to maximize yield by identifying the optimal combination of variable levels within the tested ranges. The model's validity was confirmed through three verification experiments under the predicted optimal conditions. For comparative analysis, the DES solvent was replaced with water, and extractions were performed under the same optimized parameters (with necessary adjustments). This produced polysaccharide samples from the pericarp (ZBP-H) and leaves (ZBLP-H), respectively. It should be clarified that this control method is referred to as “ultrasound-assisted water extraction” throughout the text, which differs from conventional hot-water extraction without ultrasound assistance. The samples were stored at 4 °C for subsequent use.

### 2.6 Chemical structure determination of ZBPs and ZBLPs

#### 2.6.1 Analysis of monosaccharide composition

The monosaccharide composition of the polysaccharides was analyzed using the 1-phenyl-3-methyl-5-pyrazolone (PMP) pre-column derivatization method followed by high-performance liquid chromatography (HPLC), with slight modifications based on the procedure by Sun et al. (2014). In summary, 20 mg of the polysaccharide sample was hydrolyzed with 8 mL of 2 mol/L trifluoroacetic acid (TFA) at 110 °C for 5 h. After cooling to room temperature, the hydrolysate was centrifuged at 6000 r/min for 10 min. The supernatant was collected, mixed with methanol, and evaporated to dryness under reduced pressure at 60 °C using a rotary evaporator. This step was repeated twice to ensure complete removal of residual TFA. The dried hydrolysate was then reconstituted in 0.2 mL of deionized water. To this solution, 0.2 mL of 0.3 mol/L sodium hydroxide and 0.2 mL of 0.5 mol/L PMP-methanol solution were added. The mixture was vortexed and incubated at 70 °C for 100 min in the dark. After cooling, the reaction was quenched by neutralizing with 0.2 mL of 0.3 mol/L hydrochloric acid to adjust the pH to 7.0 ± 0.1, as confirmed by pH indicator paper (pH range 0–14, precision 0.1 pH unit). The pH was carefully monitored to ensure complete neutralization of the residual TFA and alkaline reaction mixture, as excessive acidity or alkalinity could affect the stability of the PMP-derivatized monosaccharides. The resultant solution was extracted three times with 1 mL of chloroform to remove excess PMP reagent. The final aqueous phase was collected and diluted to a volume of 5 mL with deionized water. Before HPLC injection, an aliquot of the solution was filtered through a 0.22 μm organic phase membrane filter. A standard monosaccharide mixture, including rhamnose, mannose, galacturonic acid, glucuronic acid, galactose, glucose, xylose, and arabinose (each at 0.5 mg/mL), was derivatized using the same procedure. HPLC analysis was conducted on an Agilent 1260 series system equipped with a UV detector (Agilent Technologies, Santa Clara, CA, USA). Separation was achieved using a PHENOMENEX C18 column (250 mm × 4.6 mm, 5 μm) maintained at 30 °C. The mobile phase consisted of acetonitrile and 0.05 mol/L phosphate buffer (pH 6.8) (17.5:82.5, *v*/v) delivered at a flow rate of 1.0 mL/min. The detection wavelength was set at 245 nm, and the injection volume was 10 μL.

#### 2.6.2 Fourier transform infrared (FT-IR) spectroscopy analysis

A polysaccharide sample weighing approximately 2.0 mg was thoroughly mixed with dried potassium bromide (KBr) powder and ground to a fine consistency. This mixture was then pelletized under pressure at 25 °C. To serve as a background reference, a pure KBr pellet weighing 200.0 mg was prepared under the same conditions. FT-IR spectra were obtained using a Thermo Nicolet Nexus 870 ESP spectrometer (Carlsbad, CA, USA) across the spectral range of 4000–400 cm^−1^.

### 2.7 Antioxidant capacity assaysis

The antioxidant activities of ZBP-D, ZBP-H, ZBLP-D, and ZBLP-H were determined to evaluate the impact of DES extraction on the bioactivities of polysaccharides. The scavenging capacities for DPPH, ABTS, and hydroxyl radicals were measured using commercial assay kits, following the manufacturer's instructions.

### 2.8 Preparation, microstructural characterization, and property evaluation of ZB polysaccharide hydrogels

Blank hydrogels (BLK group), as well as *ZB* pericarp and leaf polysaccharide hydrogels (designated as ZBP hydrogels and ZBLP hydrogels, respectively), were prepared according to the method described by Liu et al. with slight modifications (Liu et al., 2022). A 3% (*w*/*v*) chitosan solution in acetic acid and a 3% (*w*/*v*) citric acid solution were mixed at a volume ratio of 2:1. After thorough mixing, an equal volume of *ZB* polysaccharide solution at a concentration of 4000 μg/mL was added, and the mixture was vigorously shaken, then frozen at −20 °C for 24 h. After removal, it was thawed at room temperature for approximately 1 h to obtain the *ZB* polysaccharide hydrogel. A single freeze-thaw cycle was performed; no repeated freeze-thaw cycles were applied. The blank hydrogel was prepared in the same manner, except that the polysaccharide solution was replaced with an equal volume of deionized water. The obtained hydrogels were freeze-dried at −60 °C. The cross-sections of the samples were subjected to vacuum gold-sputter coating using a Hitachi E-1010 ion sputter coater at 10 mA for 60 s under vacuum. The coated samples were observed using a scanning electron microscope (SEM; S-3400 N, Hitachi Ltd., Tokyo, Japan) at an accelerating voltage of 5 kV with a magnification of 100×. Pore size analysis was performed using Nano Measurer (version 1.2; Department of Chemistry, Fudan University, Shanghai, China). For each hydrogel group, 2–3 representative SEM images (100× magnification) were selected. From these images, 15 structurally intact pores with clear edges in the central region were randomly selected and measured (*n* = 15 per group). To ensure comparability across all hydrogel groups, a unified measurement strategy was applied, with the same number of pores measured for each group. The blank hydrogel exhibited a sparse and heterogeneous pore structure; therefore, only structurally intact pores with clear boundaries were selected for measurement.

The swelling rate, water-holding capacity, and loading rate of the hydrogels were determined following the method described by Chen et al. (2023). The hemolysis ratio was determined following the method of Zheng et al. (2021). Antioxidant activity was assessed according to the protocols outlined in Section 2.7, using 200 μg of lyophilized polysaccharide hydrogel powder (including ZBP and ZBLP hydrogels) as the test sample; the BLK group was similarly evaluated. The scavenging capacities against DPPH, ABTS, and hydroxyl radicals were measured.

### 2.9 Statistical analysis

All experiments were conducted in triplicate, with results expressed as mean ± standard deviation. Statistical analyses utilized IBM SPSS Statistics (version 28.0.1.1; IBM Corp., Armonk, NY, USA, 2021), applying one-way analysis of variance (ANOVA) followed by Duncan's test to evaluate significance, with a *P* value of less than 0.05 deemed statistically significant. The response surface methodology experimental design and its statistical analysis were carried out using Design-Expert (version 13.0.1.0, Stat-Ease Inc., Minneapolis, MN, USA, 2021).

## 3 Results and discussion

### 3.1 Screening of DESs

As a novel class of green solvents, DESs have become widely used for extracting plant polysaccharides. A key advantage of DESs is their tunable physicochemical properties, which can be precisely adjusted by modifying the chemical composition and molar ratio of their components (Ling & Hadinoto, 2022). These changes directly affect the hydrogen-bonding capacity and, consequently, the extraction efficiency. Choline chloride and betaine, known for their biodegradability and excellent properties, are widely recognized as effective HBAs for DES synthesis (Zdanowicz et al., 2018). In this study, twelve different DESs were designed based on systems previously reported for extracting other polysaccharides. These DESs were evaluated to determine the most suitable one for extracting ZBPs and ZBLPs. As illustrated in Fig. 1A and B, the extraction efficiency varied significantly among the different DES types. DES1, composed of choline chloride and oxalic acid, achieved the highest extraction rates for both ZBPs and ZBLPs. This superior performance is likely due to stronger electrostatic interactions between DES1 and the target polysaccharides compared to other DESs.

**Fig. 1:**
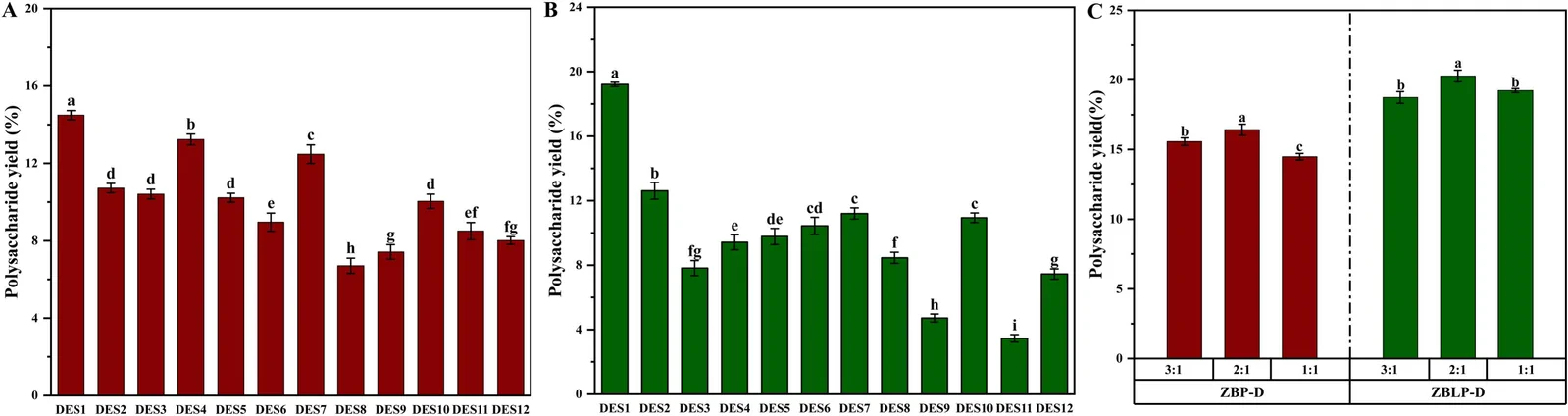
(A) Effects of different DES systems on the extraction yield of ZBP-D. (B) Effects of different DES systems on the extraction yield of ZBLP-D. (C) Effects of different molar ratios of HBA to HBD on the extraction yields of ZBP-D and ZBLP-D. Bars represent standard deviations of three independent replicates. Different letters above the columns indicate significant differences (*P* < 0.05).

The choline chloride-oxalic acid DES system (2:1 M ratio) selected as the optimal solvent has been partly characterized in prior studies. Recent investigations have reported the densities, viscosities, and structural features of choline chloride/oxalic acid DES confirmed by ^1^H NMR and FTIR spectroscopy (Sibiya et al., 2024). The physicochemical properties of hydrophilic DESs based on choline chloride and oxalic acid, including density, viscosity, and FTIR spectral comparison, have also been systematically evaluated (Martín et al., 2024). Furthermore, in-depth studies on choline chloride-dicarboxylic acid DES systems have reported density, viscosity, conductivity, and X-ray diffraction profiles, with molecular dynamics simulations revealing that charged species are held firmly by acid molecules through hydrogen-bonding interactions (Gontrani et al., 2019). The effect of water on the properties of choline chloride-carboxylic acid DESs has also been systematically investigated, demonstrating that water is gradually incorporated into the DES structure without its decomposition, and that properties such as density, viscosity, and conductivity vary predictably with water content (Ninayan et al., 2023). However, these literature data were obtained under conditions that differ from those of the present study, particularly regarding the presence of 30% water. Nevertheless, it should be noted that the characterization data presented in this study focus on the extraction performance and the properties of the recovered polysaccharides, rather than on a comprehensive physicochemical characterization of the DES system itself. The specific viscosity and pH values of the aqueous DES system (with 30% water) under our experimental conditions may differ from literature reports on anhydrous or lower-water-content systems, and these parameters represent important directions for future investigation, along with systematic recyclability testing and residual analysis of choline chloride and oxalic acid in the final products.

Additionally, choline chloride is generally recognized as safe (GRAS) and is commonly used in food and pharmaceutical applications. Regarding oxalic acid, although it is a naturally occurring organic acid, its residual levels in food and biomedical products must be carefully considered due to its ability to form oxalates. In this study, the polysaccharides were subjected to ethanol precipitation, centrifugation, and freeze-drying, which are expected to significantly reduce residual DES components. Nonetheless, the choline chloride-oxalic acid DES demonstrated the highest extraction efficiency and was therefore selected for subsequent experiments.

The molar ratio between the HBD and HBA is a crucial factor in determining the hydrogen-bonding interactions within a DES, which subsequently affects its viscosity and surface tension, ultimately influencing the efficiency of polysaccharide extraction (El Achkar et al., 2021). Notably, crystallization of the DES can occur at molar ratios of ≤1:2 or ≥3:1. To investigate this, choline chloride-oxalic acid (DES1) was prepared at molar ratios of 3:1, 2:1, and 1:1. As shown in Fig. 1C, the extraction yields of ZBPs and ZBLPs were significantly impacted by the molar ratio of DES1, with the highest yield observed at a 2:1 ratio. Thus, this optimal ratio was chosen for all subsequent extractions using DES1.

At the initial screening stage, polysaccharide recovery was used as the primary evaluation criterion to identify the most effective DES system among the twelve candidates. This approach is consistent with the typical practice in DES screening studies, where yield serves as the direct indicator of extraction performance. Subsequently, the selected DES (DES1, choline chloride-oxalic acid 2:1) was subjected to systematic optimization of extraction conditions (Section 3.2) and comparative evaluation with water-based ultrasound-assisted extraction, including both extraction yields and antioxidant activities (Sections 3.2.2 and 3.4).

### 3.2 Optimization of the extraction procedure by DESs

#### 3.2.1 Single-factor experiments

In ultrasound-assisted extraction using DES, ET, ED, and SSR were identified as critical parameters influencing polysaccharide yield. Initial single-factor experiments were performed to examine their individual effects and establish suitable ranges for further optimization.

The effect of ET on extraction efficiency is depicted in Fig. 2A. As the temperature increased from 40 to 80 °C, polysaccharide yields consistently rose. This trend is due to the decreased viscosity and surface tension of the DES at higher temperatures, which enhance mass transfer and polysaccharide solubility. However, beyond 80 °C, the yield began to decline, likely because of weakened solvent–solute interactions and possible degradation of heat-sensitive polysaccharides (Cvjetko Bubalo et al., 2018; Fu, Wang, Belwal, Xu, et al., 2021). Therefore, 80 °C was selected as the optimal ET. Fig. 2B illustrates that polysaccharide yield initially increased with prolonged ED up to 60 min, reaching a maximum for both ZBPs and ZBLPs. The increase from 20 to 60 min can be attributed to enhanced cell wall disruption and improved release of polysaccharides. Extending extraction time beyond 60 min, however, led to a decline in yield, presumably due to polysaccharide degradation under prolonged ultrasonic treatment (Wu et al., 2021). Consequently, 60 min was chosen as the optimal ED. The influence of SSR on extraction yield is shown in Fig. 2C. Increasing SSR from 10:1 to 80:1 mL/g resulted in an initial rise followed by a decrease in polysaccharide recovery. The highest yield was observed at an SSR of 70:1 mL/g. This pattern suggests that a moderate SSR favors polysaccharide dissolution by strengthening the concentration gradient and enlarging the contact area. Beyond 70:1 mL/g, the dilution of the solvent system may approach a saturation point, reducing extraction efficiency and potentially co-extracting impurities (Sukor et al., 2020). Hence, an SSR of 70:1 mL/g was adopted. In summary, the single-factor experiments identified the following preliminary optimal conditions: ET of 80 °C, ED of 60 min, and SSR of 70:1 mL/g for subsequent optimization steps.

**Fig. 2:**
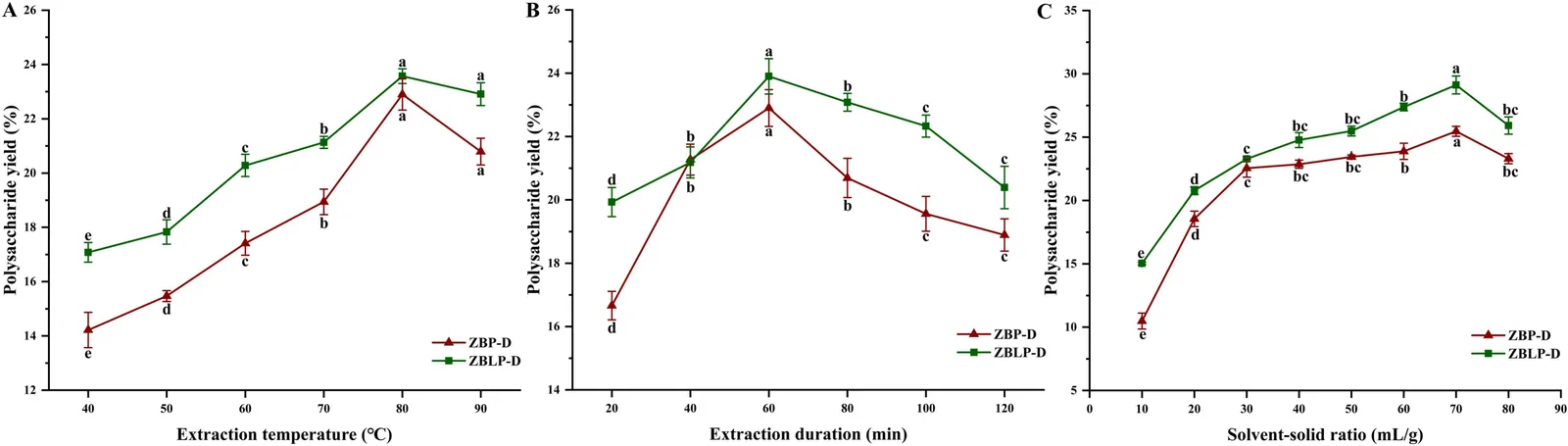
Effects of a single factor on the polysaccharide yield. (A) ET, (B) ED, (C) SSR. Bars represent the standard deviation of three individual samples. Different letters indicate significant differences (*P* < 0.05).

#### 3.2.2 Model fitting and response surface analysis

The ultrasound-assisted DES extraction process for polysaccharides was optimized using RSM. Building on preliminary single-factor experiments, a BBD with 17 experimental runs was conducted (Table S1 and Table S2). The extraction yields of ZBP-D and ZBLP-D are represented by the following second-order polynomial equations: Y₁ and Y₂ denote the predicted extraction yields of ZBP-D and ZBLP-D, respectively, while A, B, and C correspond to the independent variables ET, ED, and SSR.

(1) $$\begin{array}{c} Y_{1}=-354.99625+6.13043A+1.4089\text{0B}+2.5435\text{7C}-0.006962\mathrm{AB}-0.013300\mathrm{AC}\\ -0.00163\mathrm{BC}-0.029355A^{2}-0.006808B^{2}-0.010505C^{2} \end{array}$$

(2) $$\begin{array}{c} Y_{2}=-147.30875+1.84982A+0.622537B+2.33355C+0.000600\mathrm{AB}-0.003175\mathrm{AC}\\ -0.002113\mathrm{BC}-0.009795A^{2}-0.004524B^{2}-0.014120C^{2} \end{array}$$

Significant correlations between the response values (polysaccharide yields) and the three independent variables are revealed in Table 2 and Table 3. The F-values indicate that the order of influence on ZBP-D yield is ET > ED > SSR, mirroring the order for ZBLP-D yield. Both regression models are highly significant, supported by high F-values (ZBP-D: F = 130.31; ZBLP-D: F = 83.00) and extremely low *P*-values (*P* < 0.0001). Additionally, the lack-of-fit terms for both models are not significant (*P* > 0.05), affirming the models' goodness-of-fit and reliability. ANOVA results show that the quadratic terms (A^2^, B^2^, C^2^) are highly significant (*P* < 0.01) in both models. For the ZBP-D model, the coefficient of determination (R₁^2^) is 0.9941, and the adjusted R₁^2^ (Adj R₁^2^) is 0.9864, indicating that the model accounts for 98.64% of the response variability and is highly reliable for predicting ZBP-D extraction yield. Similarly, the ZBLP-D model exhibits high fitting accuracy, with R₂^2^ and Adj R₂^2^ values of 0.9907 and 0.9788, respectively, demonstrating its strong predictive capability for subsequent experimental outcomes.

**Table 2:** ANOVA for the ZBP-D response surface quadratic model.

Source	Sum of squares	*df*	Mean square	F-value	*P*-value	Significance
Model	101.52	9	11.28	130.31	<0.0001	⁎⁎
A	5.76	1	5.76	66.58	<0.0001	⁎⁎
B	1.79	1	1.79	20.63	0.0027	⁎⁎
C	0.0006	1	0.0006	0.0071	0.9353	
AB	7.76	1	7.76	89.60	<0.0001	⁎⁎
AC	7.08	1	7.08	81.74	<0.0001	⁎⁎
BC	0.0042	1	0.0042	0.0488	0.8315	
A^2^	36.28	1	36.28	419.15	<0.0001	⁎⁎
B^2^	31.22	1	31.22	360.66	<0.0001	⁎⁎
C^2^	4.65	1	4.65	53.68	0.0002	⁎⁎
Residual error	0.6059	7	0.0866			
Lack of fit	0.2178	3	0.0726	0.7485	0.5774	
Pure error	0.3881	4	0.0970			
Cor total	102.12	16				
R_1_^2^	0.9941					
Adj R_1_^2^	0.9864					
C.V.%	1.31					

⁎⁎ Highly significant (*P* < 0.01).

**Table 3:** ANOVA for the ZBLP-D response surface quadratic model.

Source	Sum of squares	*df*	Mean square	F-value	*P*-value	Significance
Model	39.43	9	4.38	83.00	<0.0001	⁎⁎
A	7.43	1	7.43	140.77	<0.0001	⁎⁎
B	1.30	1	1.30	24.71	0.0016	⁎⁎
C	0.4608	1	0.4608	8.73	0.0213	⁎
AB	0.0576	1	0.0576	1.09	0.3309	
AC	0.4032	1	0.4032	7.64	0.0279	⁎
BC	0.7140	1	0.7140	13.53	0.0079	⁎⁎
A^2^	4.04	1	4.04	76.53	<0.0001	⁎⁎
B^2^	13.79	1	13.79	261.18	<0.0001	⁎⁎
C^2^	8.39	1	8.39	159.04	<0.0001	⁎⁎
Residual error	0.3695	7	0.0528			
Lack of fit	0.2856	3	0.0952	4.54	0.0891	
Pure error	0.0839	4	0.0210			
Cor total	39.80	16				
R_1_^2^	0.9907					
Adj R_1_^2^	0.9788					
C.V.%	0.8365					

⁎ Significant (*P* < 0.05).

⁎⁎ Highly significant (*P* < 0.01).

The interactive effects of the three factors on polysaccharide extraction efficiency are depicted through three-dimensional (3D) response surface plots and their corresponding contour plots (Fig. 3). A steeper curvature in the 3D plot and a more elliptical shape in the contour plot generally indicate a stronger influence of the respective factor on polysaccharide yield (Zhang & Wang, 2017). As illustrated in Fig. 3A and B, the interaction effects of AB (ET × ED) and AC (ET × SSR) were highly significant (*P* < 0.01). In contrast, the BC (ED × SSR) interaction was not significant (Fig. 3C). The model predicted optimal extraction conditions as ET = 81.57 °C, ED = 60.96 min, and SSR = 68.96 mL/g, with a predicted yield of 25.66%. For practical experimental feasibility, these parameters were adjusted to ET = 82 °C, ED = 61 min, and SSR = 69 mL/g. Under these modified conditions, the actual yield of ZBP-D was 25.88% ± 0.54% (*n* = 3), closely aligning with the predicted value. For comparison, using deionized water as the extraction solvent under the same conditions resulted in a ZBP-H yield of only 6.27% ± 0.24%. These results demonstrate a 4.13-fold increase in extraction efficiency of ZBPs using the DES-based method compared to the traditional aqueous method.

**Fig. 3:**
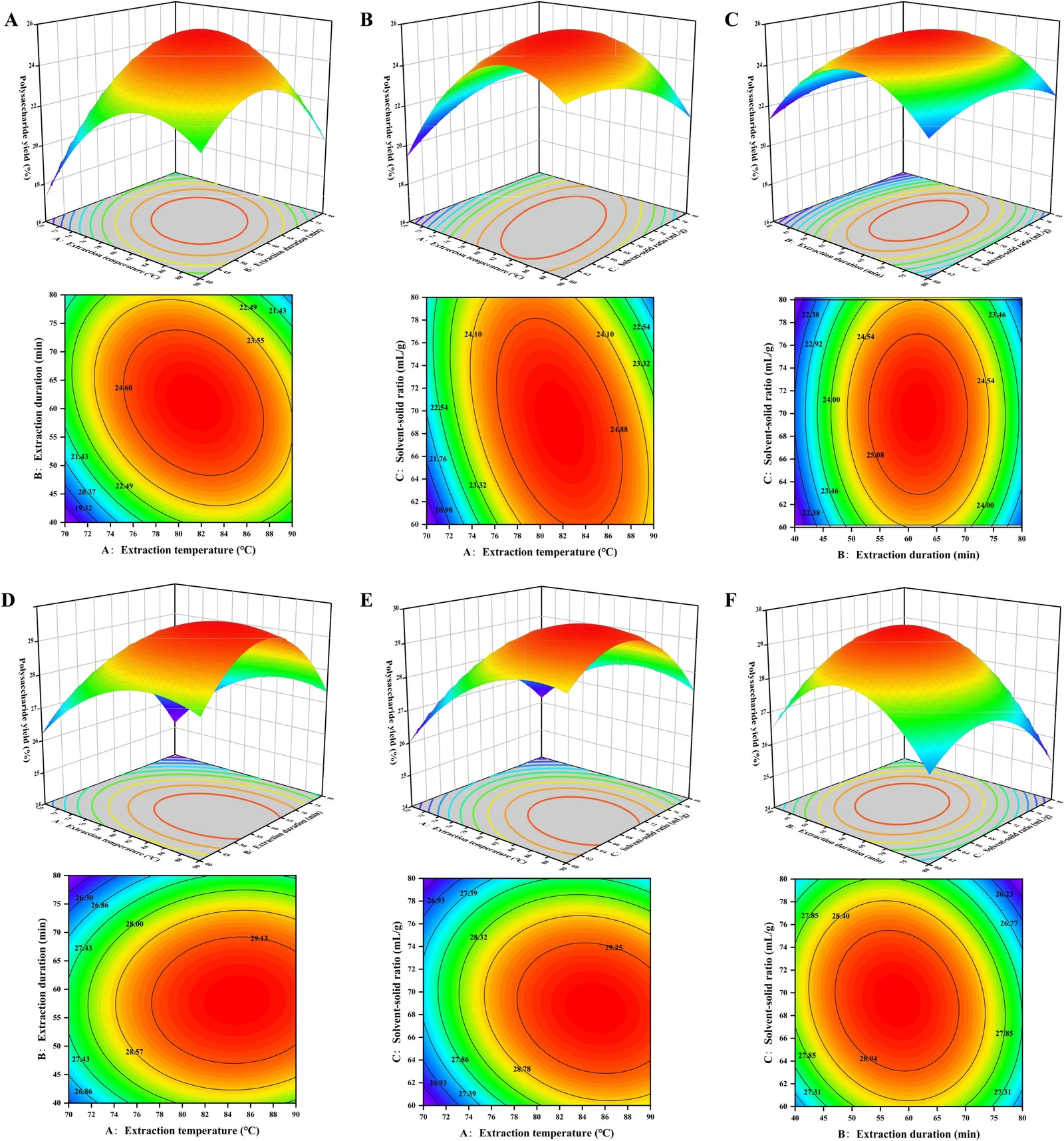
Response surface and contour plots illustrating factor interactions on polysaccharide yield. (A-C) ZBP-D: (A) ET × ED, (B) ET × SSR, (C) ED × SSR. (D—F) ZBLP-D: (D) ET × ED, (E) ET × SSR, (F) ED × SSR. ET: extraction temperature; ED: extraction time; SSR: solvent-to-solid ratio.

As shown in Fig. 3D, E and F, the interactive effects of the three factors on ZBLP-D yield were assessed. The study found no statistically significant interaction between ET and ED (AB). However, significant correlations were observed between ET and SSR (AC) and between ED and SSR (BC). The model predicted optimal extraction conditions as ET = 90 °C, ED = 58.87 min, and SSR = 68.12 mL/g, resulting in a ZBLP-D yield of 29.49%. For practical application, the parameters were adjusted to ET = 90 °C, ED = 59 min, and SSR = 68 mL/g. Under these conditions, the actual ZBLP-D yield was 29.79% ± 0.05% (*n* = 3), closely matching the predicted value. In comparison, using deionized water as the extraction solvent under the same conditions yielded only 12.13% ± 0.83% of ZBLP-H. This demonstrates that the DES-based extraction method enhanced the extraction efficiency of ZBLPs by 2.46 times compared to the traditional aqueous method.

These results collectively demonstrate that the experimental yields of ZBP-D and ZBLP-D align well consistency with the model-predicted values. Moreover, compared to the previously reported ultrasound-assisted water extraction method for ZBPs, which had an optimal yield of 13.96 ± 0.84%, the DES-based approach offers clear advantages. These include a shorter extraction time and significantly higher extraction efficiency (Li et al., 2015).

The enhanced extraction efficiency of the choline chloride-oxalic acid DES system can be attributed to multiple synergistic mechanisms. The strong hydrogen-bonding capacity of DES facilitates the disruption of the plant cell wall polysaccharide network by competing with the inter- and intramolecular hydrogen bonds among cellulose, hemicellulose, and pectin components (Sauvetes et al., 2026). In parallel, the acidic nature of oxalic acid (the HBD component) promotes the hydrolysis of glycosidic linkages in cell wall structural polysaccharides, thereby increasing the release of target polysaccharides. Another contributing factor is the relatively low viscosity of the DES-water system (30% water, *w*/w) under the optimized extraction temperatures (80–90 °C), which enhances mass transfer and solvent penetration into the plant matrix. Superimposed on these chemical and physical effects, ultrasound assistance further promotes cell wall disruption through cavitation and mechanical effects, synergistically improving the extraction yield (Li et al., 2024). This mechanistic understanding is consistent with previous reports on DES-based extraction of plant polysaccharides, which have demonstrated that choline chloride-oxalic acid DES exhibits superior extraction performance due to its favorable physicochemical properties (Luo et al., 2024).

The higher extraction yield of leaf polysaccharides (29.79%) compared to pericarp polysaccharides (25.88%) may be attributed to differences in tissue structure. Fruit pericarps generally possess more rigid cell walls with higher lignin deposition to serve a protective function, whereas leaf tissues typically have thinner, less lignified primary walls that facilitate metabolic exchange. This structural disparity likely renders leaf cell walls more susceptible to DES-mediated disruption, thereby contributing to the observed higher polysaccharide yield from leaves. A comprehensive cell wall compositional analysis of the raw materials was beyond the scope of this study and is identified as an important direction for future investigation.

### 3.3 Chemical structure determination of ZBPs and ZBLPs

#### 3.3.1 Monosaccharide composition analysis

Monosaccharide composition is crucial for understanding the primary structure of polysaccharides. In this study, the monosaccharide profiles of four extracted polysaccharides—ZBP-D, ZBP-H, ZBLP-D, and ZBLP-H—were analyzed. As illustrated in Fig. 4 and Fig. 5A, all four polysaccharides displayed similar monosaccharide types, including mannose (Man), rhamnose (Rha), glucuronic acid (GlcA), galacturonic acid (GalA), glucose (Glc), galactose (Gal), xylose (Xyl), and arabinose (Ara). Previous research indicates that the monosaccharide composition of ZBPs and ZBLPs is not always uniform. For instance, Chen et al. (2021) identified six monosaccharides in ZBPs, with GalA being the most prevalent component. In contrast, Sang et al. (2025) found that ZBLPs were primarily composed of Man, GalA, Glc, Gal, and Ara. These differences in monosaccharide composition between ZBPs and ZBLPs may result from factors such as plant variety, geographical origin, climatic conditions, and extraction methods. In the present work, the major monosaccharides in both ZBPs and ZBLPs obtained through different extraction methods were consistently GalA, Glc, Gal, and Ara, though their molar ratios differed. Among these, Glc and Gal were the most abundant monosaccharides in all four polysaccharide samples, suggesting that both types of polysaccharides contain significant amounts of arabinogalactan and glucan.

**Fig. 4:**
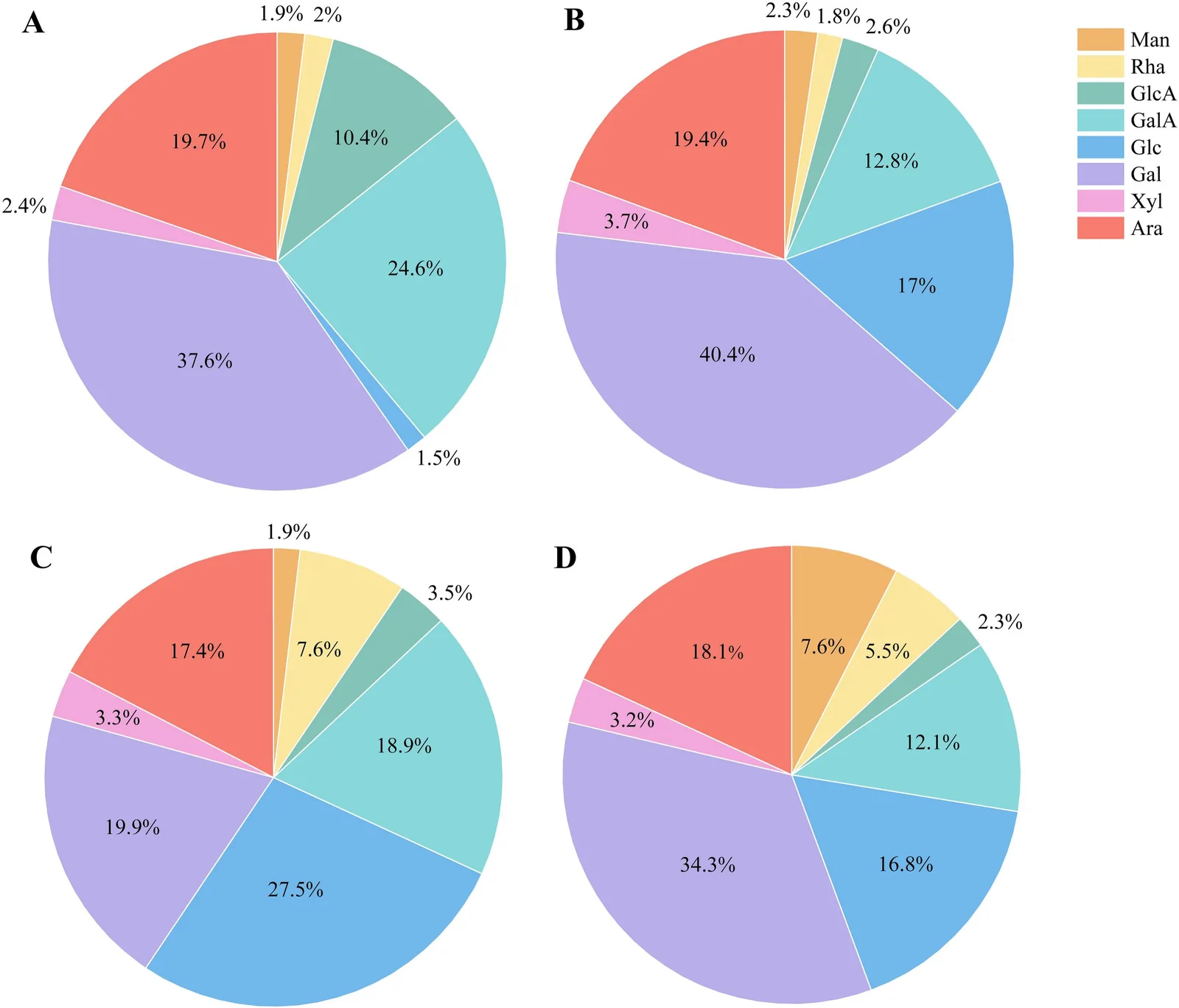
The monosaccharide composition of ZBPs and ZBLPs. (A) ZBP-D, (B) ZBP-H, (C) ZBLP-D, (D) ZBLP-H.

**Fig. 5:**
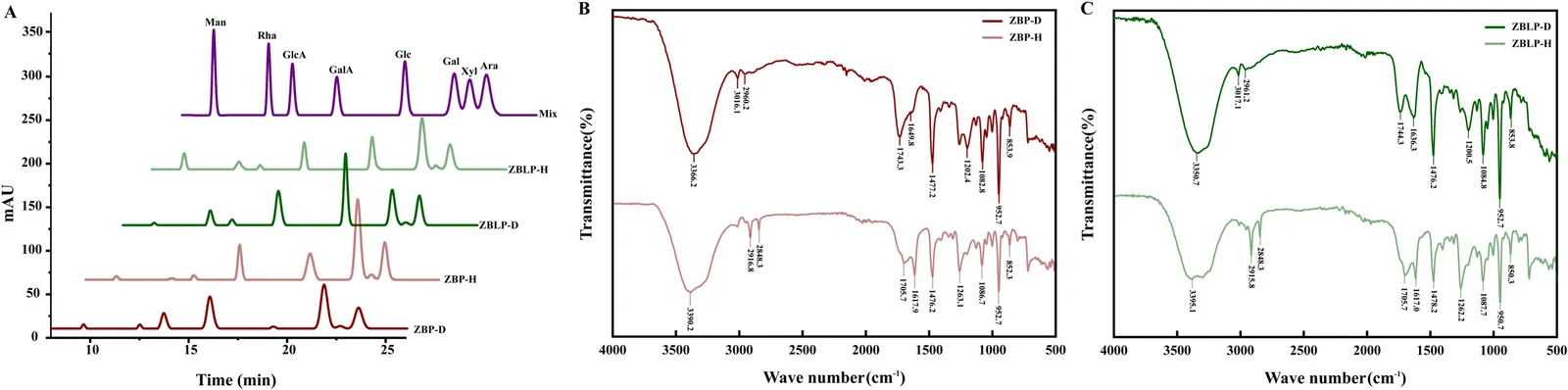
Comparative structural characterization of ZBPs and ZBLPs. (A) HPLC analysis of monosaccharide composition, (B) FT-IR spectra of ZBP-D and ZBP-H, (C) FT-IR spectra of ZBLP-D and ZBLP-H.

#### 3.3.2 FT-IR spectra analysis

FT-IR spectroscopy was utilized to identify the characteristic functional groups within polysaccharide structures. This technique enables qualitative analysis of functional groups and chemical bonds through their specific infrared absorption peaks, thereby confirming the presence of typical polysaccharide features (Chen, Wang, et al., 2019). The FT-IR spectra for ZBP-D, ZBLP-D, ZBP-H, and ZBLP-H are displayed in Fig. 5A and Fig. 5B, respectively. All four polysaccharides showed similar spectral patterns, with only slight variations in absorption intensities. A broad and smooth absorption peak around 3600–3200 cm^−1^ was attributed to O—H stretching vibrations, indicating intra- and intermolecular hydrogen bonds (Li et al., 2021). These hydroxyl groups contribute to the antioxidant activity of polysaccharides by acting as electron donors. The absorption bands in the region of 3000–2800 cm^−1^ were assigned to C—H stretching vibrations, characteristic of polysaccharides (Nie et al., 2018). A peak near 1740 cm^−1^ resulted from the C=O stretching of esterified carboxyl groups (-COOCH₃), suggesting the presence of sugar acids (Chen, Zhang, et al., 2019). In contrast, the absorption around 1620 cm^−1^ corresponded to the C=O stretching of non-esterified carboxyl groups (-COOH) (Li et al., 2021). The absorption bands between 1400 and 1200 cm^−1^ were linked to C—H bending vibrations (Chen, Wang, et al., 2019). The absorption band in the 1200–950 cm^−1^ region corresponds to characteristic stretching vibrations of C-O-C and C-O-H linkages, confirming the presence of pyranose configurations. These spectral features are particularly indicative of β-glucan structures, as evidenced by the distinctive patterns associated with O-substituted glucose residues (Fan et al., 2017; Wang et al., 2015). Additionally, the spectral band observed at 850 cm^−1^ confirms the existence of α-configurational sugar units in both ZBPs and ZBLPs (Liu et al., 2014).

It should be acknowledged that the structural characterization presented in this study is limited to monosaccharide composition and FT-IR analysis. While these techniques confirm the typical polysaccharide features and reveal differences in monosaccharide molar ratios between DES- and ultrasound-assisted water-extracted samples, they are insufficient to fully establish detailed structural similarity or comprehensive structure-activity relationships. Key structural parameters such as molecular weight distribution, glycosidic linkage types (e.g., 1 → 4, 1 → 6 linkages), chain conformation, and degree of branching, all of which are known to significantly influence the bioactivities of polysaccharides, were not determined in the current study. Therefore, the conclusion that the polysaccharides share “similar structures” is based on the comparable functional groups and monosaccharide types observed, rather than on complete structural elucidation. Advanced techniques including HPSEC-MALLS for molecular weight determination, methylation analysis for glycosidic linkage identification, NMR spectroscopy for detailed structural resolution, and Congo red assay for conformational analysis are required to provide a more comprehensive structural characterization in future investigations.

### 3.4 Antioxidant capacity analysis

The polysaccharides ZBP-D, ZBLP-D, ZBP-H, and ZBLP-H demonstrated dose-dependent scavenging activities against DPPH, ABTS, and hydroxyl radicals. As shown in Fig. 6A, both ZBP-D and ZBLP-D exhibited stronger DPPH radical scavenging capabilities than ZBP-H and ZBLP-H. Conversely, the ABTS radical scavenging ability of ZBP-D and ZBLP-D was lower than that of their ultrasound-assisted water extracted variants (Fig. 6B). Notably, ZBP-D and ZBLP-D displayed significantly enhanced hydroxyl radical scavenging activity, with IC₅₀ values of 0.231 mg/mL and 0.513 mg/mL, respectively, which were substantially lower than those of ZBP-H (2.385 mg/mL) and ZBLP-H (2.701 mg/mL) (Fig. 6C). At a concentration of 2.0 mg/mL, the hydroxyl radical scavenging rates of ZBP-D and ZBLP-D reached 95.46 ± 0.61% and 91.07 ± 0.04%, respectively. The IC₅₀ values for DPPH, ABTS, and hydroxyl radical scavenging activities of all samples are summarized in Table 4.

**Fig. 6:**
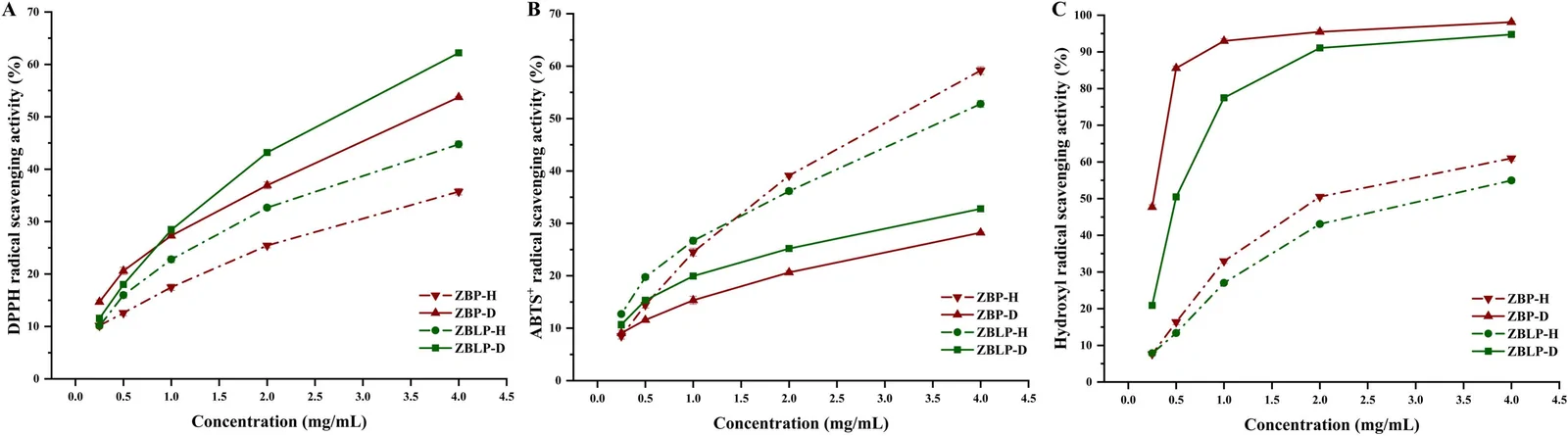
Antioxidant activities of ZBPs and ZBLPs. (A) DPPH radical scavenging activity, (B) ABTS radical scavenging activity, (C) hydroxyl radical scavenging activity.

**Table 4:** IC₅₀ values (mg/mL) of ZBPs and ZBLPs for DPPH, ABTS, and hydroxyl radical scavenging activities.

Category	IC_50_(mg/mL)		
	DPPH	ABTS^+^	-OH
ZBP-D	3.828 ± 0.074c	15.464 ± 0.777b	0.231 ± 0.003d
ZBP-H	10.314 ± 0.581a	2.408 ± 0.040d	2.385 ± 0.059b
ZBLP-D	2.553 ± 0.053d	18.575 ± 0.534a	0.513 ± 0.004c
ZBLP-H	5.063 ± 0.160b	3.83 ± 0.030c	2.701 ± 0.034a

The antioxidant properties of polysaccharides are intricately linked to their chemical structures, with uronic acids content being a crucial factor (Nie et al., 2020). As illustrated in Fig. 4, the glucuronic acid (GlcA) to galacturonic acid (GalA) ratios in ZBP-D and ZBLP-D exceed those in their respective ultrasound-assisted water-extracted samples (ZBP-H/ZBLP-H). This indicates that DES extraction may selectively enrich uronic acid-rich polysaccharide fractions by preferentially solubilizing acidic polysaccharide components. The free carboxyl groups (-COOH) on uronic acid residues can enhance the release of hydrogen atoms from O—H bonds under acidic conditions, thereby boosting the hydrogen-donating capacity of polysaccharides to effectively terminate free radical chain reactions. Notably, under acidic conditions, electrophilic groups can expedite the release of hydrogen from O—H bonds (Chen et al., 2005). The distinct behavior in the ABTS^+^ assay, where ultrasound-assisted water-extracted samples outperformed DES-extracted ones, reflects differences in the single electron transfer (SET) mechanism that governs ABTS^+^ neutralization. Ultrasound-assisted water extraction may better preserve key structural features such as phenolic hydroxyl groups, reducing ends, and intact electron cloud systems on the polysaccharide chains, which remain unaltered by significant solvation. These preserved structures facilitate efficient electron transfer, directly neutralizing ABTS^+^ radicals. By contrast, the superior performance of DES-extracted polysaccharides in DPPH and hydroxyl radical scavenging assays suggests that the acidic DES environment may selectively enrich uronic acid-rich fractions. These fractions possess enhanced hydrogen-donating capacity, which is particularly effective for radical neutralization via hydrogen atom transfer (HAT) pathways (Yarley et al., 2021). This radical-selective activity pattern, where DES extraction favors HAT-based radicals (DPPH, ·OH) while water extraction favors SET-based radicals (ABTS^+^), is consistent with the differing chemical environments imposed by the two extraction methods. The antioxidant activity of polysaccharides is highly dependent on structural features including solubility, sugar ring structure, molecular weight, charge groups, protein moieties, and covalently linked phenolic compounds (Fernandes & Coimbra, 2023). Moreover, the antioxidant activity of polysaccharides is influenced by various structural factors: molecular weight, conformation, monosaccharide composition, and glycosidic linkage types all significantly affect their efficacy (Jiang et al., 2016). As noted by Zou et al. (2020), the content of aromatic monosaccharides like Ara, Gal, and Rha positively correlates with antioxidant activity, which aligns with the relative enrichment of these monosaccharides in the ultrasound-assisted water extracts observed in this study. Although existing research has uncovered some structure-activity relationships, the extensive variety and structural heterogeneity of natural polysaccharides mean that their antioxidant mechanisms can differ substantially.

Overall, these results demonstrate that the antioxidant activity of the extracted polysaccharides is highly dependent on both the extraction method and the specific radical assay employed. DES-extracted polysaccharides (ZBP-D and ZBLP-D) exhibited significantly enhanced scavenging activities against DPPH and hydroxyl radicals, with IC₅₀ values for hydroxyl radical scavenging as low as 0.231 mg/mL (ZBP-D) and 0.513 mg/mL (ZBLP-D). In contrast, ultrasound-assisted water-extracted samples (ZBP-H and ZBLP-H) showed superior ABTS^+^ radical scavenging activity. This differential pattern may reflect extraction-method-induced selective enrichment of particular polysaccharide subfractions with distinct electron-donating (SET) or hydrogen-transfer (HAT) mechanisms. These findings align with previous studies. For instance, Cao et al. (2024) reported that DES-extracted polysaccharides from wolfberry exhibited higher antioxidant activity, and similar radical-selective behavior has been observed in other DES-extracted polysaccharides where variations in uronic acid content and molecular conformation contribute to differential radical scavenging profiles. Therefore, ultrasound-assisted DES extraction not only increases polysaccharide yield but also selectively enhances their biological activities against specific radicals.

Additionally, the protein content before and after pancreatin treatment, the verification of deproteinization efficiency, and a systematic comparison with alternative deproteinization methods (such as the Sevag method, TCA method, or treatment with a specific protease alone) were not performed in the current study. These aspects represent important directions for future investigation to fully validate the purity and bioactivity of the recovered polysaccharides.

It should be acknowledged that the antioxidant activities measured in this study represent the combined effects of the crude polysaccharide extracts, which may contain residual DES components (choline chloride and oxalic acid), co-extracted phenolics or flavonoids, reducing sugars, or other small molecules. The potential contribution of these non-polysaccharide components to the observed radical scavenging activities cannot be completely excluded without comprehensive dialysis or purification steps. Controls such as DES blanks, oxalic acid or choline chloride solutions at equivalent concentrations, and compositional analyses including total phenolic content and reducing sugar content were not performed in the current study. Therefore, the reported activities should be interpreted as the overall bioactivity of the DES-extracted fraction rather than that of purely isolated polysaccharides. Future studies employing rigorous purification protocols (e.g., membrane dialysis, size-exclusion chromatography) and appropriate blanks/controls are needed to definitively attribute the bioactivities to the polysaccharide fractions themselves.

### 3.5 Microstructural characterization, and property evaluation of ZB polysaccharide hydrogels

#### 3.5.1 Microstructural characterization of ZB polysaccharide hydrogels

ZBP-D and ZBLP-D, which exhibited superior extraction efficiency, were selected for hydrogel preparation. SEM images showed that the blank hydrogel (BLK) displayed a sparse pore distribution with only a few intact three-dimensional networks (Fig. 7A), attributed to uneven ice crystal dispersion leading to heterogeneous sublimation during physical crosslinking (Zhang et al., 2023). In contrast, the ZBP-D and ZBLP-D hydrogels exhibited a similar but denser and more uniform pore structure with clearer pore walls, indicating that polysaccharide introduction promoted pore regularization without disrupting the network (Fig. 7B, C). This effect may be attributed to hydrogen bonding or electrostatic interactions between polysaccharides and the chitosan matrix, which enhanced network stability (Nie et al., 2026).

**Fig. 7:**
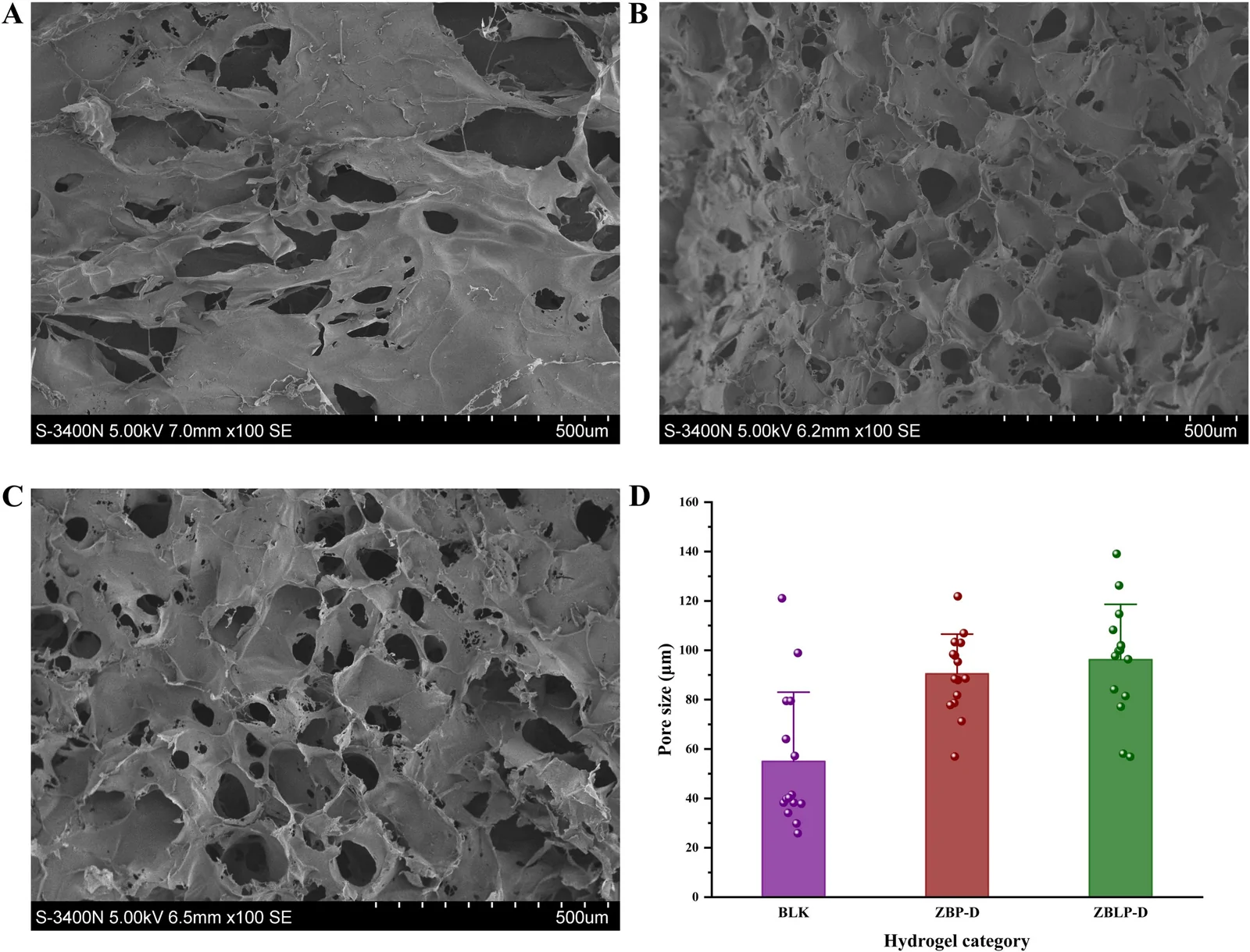
Scanning electron microscopy (SEM) images and pore size distribution diagram of the hydrogels. (A) SEM image of BLK hydrogel, (B) SEM image of ZBP-D hydrogel, (C) SEM image of ZBLP-D hydrogel, (D) Pore size distribution diagram.

Pore size distribution showed that both *ZB* polysaccharide hydrogels had significantly larger pores than the blank hydrogel, with ZBLP-D exhibiting the largest average pore size (97.6 μm) (Fig. 7D). This is likely due to the primary hydroxyl groups of polysaccharides undergoing esterification with citric acid carboxyl groups, competitively interfering with chitosan crosslinking, thereby reducing crosslinking density and expanding the pores (Zhang et al., 2023).

#### 3.5.2 Property evaluation of ZB polysaccharide hydrogels

Swelling capacity and water-holding capacity are key indicators of the moisturizing function of a hydrogel, reflecting its ability to absorb exudate and maintain a moist wound environment (Fu et al., 2025). Both ZBP-D and ZBLP-D hydrogels exhibited swelling ratios of approximately 1200% (Table 5), significantly higher than the BLK hydrogel, indicating that the introduction of *ZB* polysaccharides enhanced water absorption. The enhanced swelling ratios of ZBP-D and ZBLP-D hydrogels relative to the blank hydrogel can be attributed to the introduction of hydrophilic polysaccharide chains, which provide additional hydroxyl and carboxyl groups for water binding through hydrogen bonding. The larger pore sizes observed in polysaccharide-containing hydrogels (Fig. 7D) likely result from competitive interference of polysaccharide hydroxyl groups with citric acid crosslinking, which reduces crosslinking density and expands the pore network. This mechanism is consistent with previous observations in polysaccharide-chitosan composite hydrogels. The lower water retention rates of polysaccharide hydrogels compared to the blank hydrogel (Fig. 8A) are consistent with their larger pore sizes, which accelerate water diffusion and release.

**Table 5:** Swelling rate and loading rate of different hydrogels.

Category	Swelling rate (%)	Loading rate (%)
BLK hydrogel	773 ± 28	\
ZBP-D hydrogel	1200 ± 30	68.44 ± 0.74
ZBLP-D hydrogel	1202 ± 39	69.86 ± 0.81

**Fig. 8:**
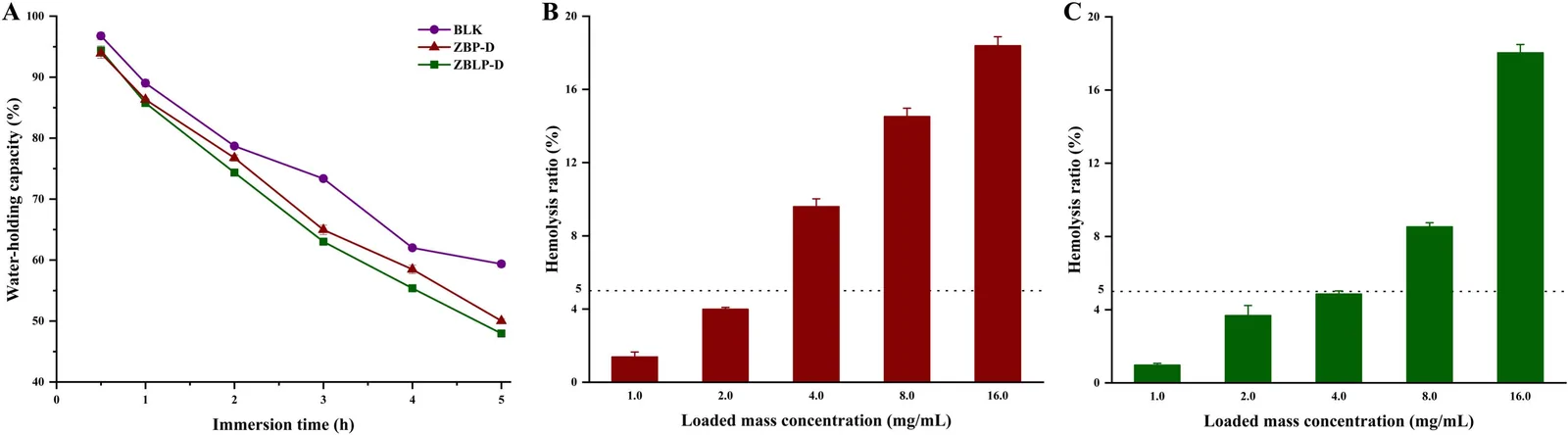
Water-holding capacity and hemolysis ratio of the hydrogels. (A) Water-holding capacity of the three hydrogels (BLK, ZBP-D and ZBLP-D), (B) Hemolysis ratio of ZBP-D hydrogel, (C) Hemolysis ratio of ZBLP-D hydrogel.

The swelling ratios of ZBP-D and ZBLP-D hydrogels (∼1200%) are comparable to those reported for other polysaccharide-based hydrogels modified with DES. For instance, pectin-chitosan eutectogels modified with choline chloride-based DES achieved maximum water absorption capacities of up to 16.02 g/g (equivalent to ∼1602%) under optimized conditions (Raffe et al., 2024). Inulin and agave fructan-based polyurethane hydrogels synthesized using choline chloride-1,4-butanediol DES exhibited tunable swelling properties suitable for drug delivery applications (Elizalde-Mata et al., 2025). Polysaccharide-based supramolecular eutectogels prepared from chitosan and chitosan/cellulose composites in DES also demonstrated significant swelling capacities, with properties influenced by hydrogen bond donor type (Marullo et al., 2023). The swelling performance of our ZBP-D and ZBLP-D hydrogels falls within the range of these reported values, confirming their favorable water absorption capacity.

Encapsulation efficiencies were 68.44 ± 0.74% for ZBP-D and 69.86 ± 0.81% for ZBLP-D (Table 5). The weakly acidic environment created by citric acid enhances electrostatic interactions between polysaccharides and the chitosan matrix, facilitating polysaccharide loading (Gao et al., 2024).

The hemolysis rate is a core safety indicator for biomaterials, with 5% as the general safety threshold: a rate below 5% indicates no erythrocyte rupture at that concentration, meeting basic safety requirements for biomedical materials (Khan et al., 2023). The maximum safe loading concentrations were 2.0 mg/mL for the ZBP-D hydrogel and 4.0 mg/mL for the ZBLP-D hydrogel (Fig. 8B, C), indicating favorable hemocompatibility under the tested conditions. However, hemolysis data alone are insufficient to fully demonstrate biomedical safety. Comprehensive biocompatibility assessment, including cytotoxicity, cell proliferation, and in vivo evaluations, is required before considering these hydrogels for biomedical applications.

At 2000 μg/mL, the antioxidant activities of the polysaccharide hydrogels (ZBP-D and ZBLP-D) were lower than those of their corresponding free polysaccharide solutions (Fig. 9). The ZBLP-D hydrogel showed the largest decreases: 12.1% for ABTS, 12.3% for DPPH, and 6.58% for hydroxyl radicals. Possible explanations include network restriction of polysaccharide chains, limited diffusion reducing local effective concentration, and crosslinking reactions modifying antioxidant groups (e.g., hydroxyl and carboxyl) via hydrogen bonding, thereby diminishing their hydrogen-donating or electron-transfer abilities (Fu et al., 2025). This activity reduction upon hydrogel formation is consistent with the general behavior of polysaccharide-based hydrogels, where covalent or physical crosslinking restricts chain mobility and reduces the accessibility of active antioxidant groups to free radicals. The blank hydrogel exhibited higher radical scavenging than the deionized water control, owing to the inherent antioxidant activity of chitosan (Riaz et al., 2020).

**Fig. 9:**
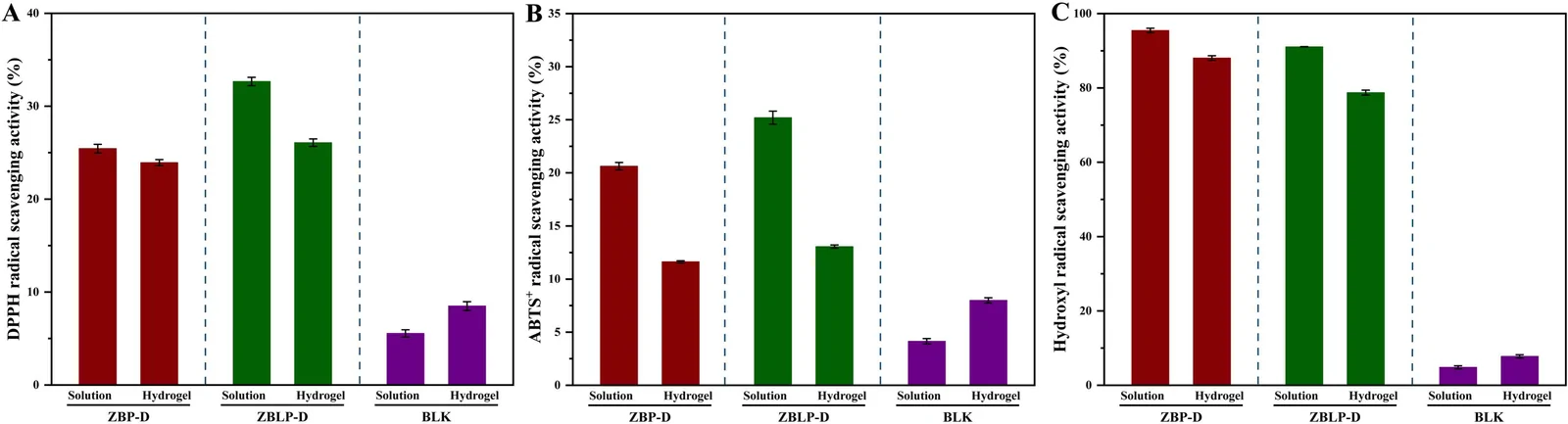
Antioxidant activity of ZBP-D and ZBLP-D solutions and their corresponding hydrogels. (A) DPPH radical scavenging activity, (B) ABTS radical scavenging activity, (C) hydroxyl radical scavenging activity.

## 4 Conclusions

In this study, a green and efficient ultrasound-assisted deep eutectic solvent (DES) extraction method was successfully developed and optimized for the recovery of polysaccharides from the pericarps and leaves of *ZB*, with the extraction performance and comparative evaluation as the primary focus. As a preliminary and exploratory extension, the extracted polysaccharides were further formulated into hydrogels to assess their basic physicochemical properties and hemocompatibility. The optimal DES system, consisting of choline chloride and oxalic acid in a 2:1 M ratio demonstrated significantly higher extraction efficiency for both ZBPs and ZBLPs compared to the conventional ultrasound-assisted water method, with yields improved by 4.13 and 2.46 times, respectively. Although monosaccharide composition and FT-IR analyses showed similar primary structures between polysaccharides extracted by DES (ZBP-D, ZBLP-D) and ultrasound-assisted water (ZBP-H, ZBLP-H), the DES-extracted polysaccharides exhibited selectively enhanced antioxidant activities: they demonstrated significantly stronger DPPH and hydroxyl radical scavenging capacities compared to their ultrasound-assisted water-extracted counterparts (with IC₅₀ values for hydroxyl radicals of 0.231 mg/mL and 0.513 mg/mL for ZBP-D and ZBLP-D, respectively). However, in the ABTS^+^ assay, the ultrasound-assisted water-extracted samples showed higher activity, indicating that the extraction method influences the radical-specific antioxidant profile of the polysaccharides. Notably, ZBP-D and ZBLP-D demonstrated excellent hydroxyl radical scavenging capacity, highlighting the unique advantage of the DES technique in obtaining polysaccharides with enhanced biological potency. More importantly, the extracted ZBP-D and ZBLP-D were successfully fabricated into functional hydrogels. These hydrogels exhibited dense three-dimensional porous networks with enlarged pore sizes (up to 97.6 μm for ZBLP-D hydrogel), high swelling ratios (∼1200%), and excellent loading ratios (68.44 ± 0.74% for ZBP-D hydrogel; 69.86 ± 0.81% for ZBLP-D hydrogel). Although the hydrogel state moderately reduced the free radical scavenging capacity relative to the free polysaccharides, the hydrogels retained considerable antioxidant activity and showed good hemocompatibility, with maximum safe loading concentrations of 2.0 mg/mL (ZBP-D) and 4.0 mg/mL (ZBLP-D), both below the 5% hemolysis safety threshold. Collectively, this work provides a sustainable, high-efficiency extraction platform for *ZB* polysaccharides and demonstrates their successful transformation into bioactive, hemocompatible hydrogels. These findings suggest that *ZB* by-products have potential for development into functional biomaterials. However, the current hydrogel data are preliminary and primarily focused on physicochemical properties and hemolytic compatibility. Comprehensive evaluations, including mechanical properties, cytotoxicity, antimicrobial activity, and in vivo wound healing efficacy, are still required before practical biomedical applications can be realized. Nevertheless, this study provides a sustainable and efficient extraction platform for *ZB* polysaccharides and offers fundamental data for their potential future development.

Several limitations of this study should be noted. Comprehensive characterization of the DES system under our specific experimental conditions, including viscosity, pH, and recyclability, was not performed; these parameters may differ from literature reports due to the addition of 30% water. The deproteinization efficiency of pancreatin was not quantitatively verified, nor was it compared with alternative methods such as Sevag or TCA treatment. Furthermore, polysaccharide structural characterization was limited to monosaccharide composition and FT-IR analysis; detailed information on molecular weight distribution, glycosidic linkage types, and chain conformation was not obtained. Future studies will address these gaps through systematic DES characterization, purification protocol optimization, and advanced structural elucidation to further validate the extraction platform and the bioactivity of the recovered polysaccharides.

## Declaration of generative AI use

We have not used any AI or AI-assisted technologies to prepare this work.

## CRediT authorship contribution statement

**Mengjia Wu:** Writing – original draft, Software, Investigation, Data curation. **Guoxing Yu:** Methodology, Data curation. **Wenya Hu:** Methodology, Data curation. **Yulin Liu:** Writing – review & editing, Investigation, Funding acquisition. **Yonghong Liu:** Writing – review & editing, Software, Investigation, Funding acquisition, Data curation.

## Declaration of competing interest

The authors declare that they have no known competing financial interests or personal relationships that could have appeared to influence the work reported in this paper.

## Data Availability

Data will be made available on request.
