# Supplementary material for: Green extraction of bioactive polysaccharides from Zanthoxylum bungeanum using deep eutectic solvents: enhanced yield, antioxidant activity, and functional hydrogel formation
Source: Food Chem X. 2026 Oct 13;39:104612. doi: 10.1016/j.fochx.2026.104612 (PMC13694982; doi:10.1016/j.fochx.2026.104612)
Supplement: Supplementary file 1 [file mmc1.docx]

| Number | A（℃） | B（min） | C（mL/g） | Y_1_（%） |
| --- | --- | --- | --- | --- |
| 1 | 80（0） | 60（0） | 70（0） | 25.57 ± 0.57 |
| 2 | 80（0） | 80（1） | 80（1） | 22.32 ± 0.30 |
| 3 | 90（1） | 40（-1） | 70（0） | 21.61 ± 0.24 |
| 4 | 80（0） | 40（-1） | 80（1） | 21.31 ± 0.60 |
| 5 | 90（1） | 60（0） | 60（-1） | 23.95 ± 0.30 |
| 6 | 80（0） | 80（1） | 60（-1） | 22.38 ± 0.50 |
| 7 | 70（-1） | 60（0） | 60（-1） | 19.29 ± 0.19 |
| 8 | 80（0） | 60（0） | 70（0） | 25.73 ± 0.59 |
| 9 | 80（0） | 60（0） | 70（0） | 25.86 ± 0.54 |
| 10 | 90（1） | 80（1） | 70（0） | 19.64 ± 0.28 |
| 11 | 80（0） | 60（0） | 70（0） | 25.06 ± 0.45 |
| 12 | 70（-1） | 40（-1） | 70（0） | 17.43 ± 0.46 |
| 13 | 70（-1） | 60（0） | 80（1） | 21.91 ± 0.30 |
| 14 | 80（0） | 60（0） | 70（0） | 25.71 ± 0.16 |
| 15 | 80（0） | 40（-1） | 60（-1） | 21.24 ± 0.21 |
| 16 | 90（1） | 60（0） | 80（1） | 21.25 ± 0.30 |
| 17 | 70（-1） | 80（1） | 70（0） | 21.03 ± 0.23 |

**Table S1**

ZBP-D response surface design program and results
