# Supplementary material for: Green extraction of bioactive polysaccharides from Zanthoxylum bungeanum using deep eutectic solvents: enhanced yield, antioxidant activity, and functional hydrogel formation
Source: Food Chem X. 2026 Oct 13;39:104612. doi: 10.1016/j.fochx.2026.104612 (PMC13694982; doi:10.1016/j.fochx.2026.104612)
Supplement: Supplementary file 2 [file mmc2.docx]

**Table S2**

ZBLP-D response surface design program and results

| Number | A（℃） | B（min） | C（mL/g） | Y_2_（%） |
| --- | --- | --- | --- | --- |
| 1 | 80（0） | 60（0） | 70（0） | 29.57 ± 0.37 |
| 2 | 90（1） | 40（-1） | 70（0） | 27.95 ± 0.08 |
| 3 | 90（1） | 60（0） | 60（-1） | 28.74 ± 0.30 |
| 4 | 80（0） | 80（1） | 60（-1） | 26.56 ± 0.35 |
| 5 | 70（-1） | 60（0） | 60（-1） | 25.98 ± 0.51 |
| 6 | 90（1） | 80（1） | 70（0） | 27.09 ± 0.15 |
| 7 | 80（0） | 60（0） | 70（0） | 29.46 ± 0.38 |
| 8 | 70（-1） | 80（1） | 70（0） | 25.12 ± 0.20 |
| 9 | 70（-1） | 60（0） | 80（1） | 26.00 ± 0.47 |
| 10 | 80（0） | 60（0） | 70（0） | 29.25 ± 0.22 |
| 11 | 80（0） | 40（-1） | 60（-1） | 26.23 ± 0.37 |
| 12 | 80（0） | 60（0） | 70（0） | 29.35 ± 0.49 |
| 13 | 80（0） | 80（1） | 80（1） | 25.37 ± 0.51 |
| 14 | 90（1） | 60（0） | 80（1） | 27.49 ± 0.12 |
| 15 | 80（0） | 60（0） | 70（0） | 29.59 ± 0.25 |
| 16 | 70（-1） | 40（-1） | 70（0） | 26.46 ± 0.49 |
| 17 | 80（0） | 40（-1） | 80（1） | 26.73 ± 0.50 |
